# Scientific support of small fruit growing in Russia and prospects for its development

**DOI:** 10.18699/VJ21.046

**Published:** 2021-07

**Authors:** I.M. Kulikov, S.N. Evdokimenko, T.A. Tumaeva, A.V. Kelina, F.F. Sazonov, N.V. Andronova, M.A. Podgaetsky

**Affiliations:** Federal Horticultural Research Center for Breeding, Agrotechnology and Nursery, Moscow, Russia; Federal Horticultural Research Center for Breeding, Agrotechnology and Nursery, Moscow, Russia; Federal Horticultural Research Center for Breeding, Agrotechnology and Nursery, Moscow, Russia; Federal Horticultural Research Center for Breeding, Agrotechnology and Nursery, Moscow, Russia; Federal Horticultural Research Center for Breeding, Agrotechnology and Nursery, Moscow, Russia; Federal Horticultural Research Center for Breeding, Agrotechnology and Nursery, Moscow, Russia; Federal Horticultural Research Center for Breeding, Agrotechnology and Nursery, Moscow, Russia

**Keywords:** scientific research institutions, small fruit crops, breeding, varieties, nursery production, certified planting material, repository, breeding and seed centers, научные исследовательские учреждения, ягодные культуры, селекция, сорта, питомниководство, сертифицированный посадочный материал, репозиторий, селекционно-семеноводческие центры

## Abstract

It is possible to achieve the target indices of the Russian Doctrine of Food Security (self-sufficiency in fruits and berries should be at least 60 %) by combining the competencies of science and business. At present, hundreds of varieties of small fruit crops are included in the State Register of Breeding Achievements Admitted for Use. Domestic breeders have obtained substantial results; the share of their assortment is 79–100 %. Federal Research Center of Horticulture (Moscow) (101 pcs.), Federal Altai Research Center of Agrobiotechnology (Barnaul) (85 pcs.), Michurin Federal Research Center (Michurinsk) (42 pcs.) are the leaders in the number of created hybrids and varieties. Over the past five years, 133 new breeding achievements of traditional small fruit crops have been submitted to the State variety testing, the originators of which are research institutions, private companies and individuals. The creation of modern seed-breeding (nursery-breeding) centers (SBC) on the basis of leading specialized research institutions is expected to be the solution to the problems of modern breeding and nursery breeding and to give impetus to the development of domestic small fruit growing. The research programs of the SBC involve an integrated approach that combines the knowledge and capabilities of researchers from different disciplines, the concentration of a complex analytical instrument base in the Centers of collective use, the using of biotechnological and molecular genetic research, along with traditional methods of breeding. An analysis of the achievements in small fruit growing in research institutions under the jurisdiction of the Ministry of Science and Higher Education of the Russian Federation revealed a huge scientific potential (genetic collections, hybrid funds) for creating competitive commercial varieties and technologies for their cultivation by establishing plantations with certified planting material in accordance with international requirements. Information from literary sources indicates that one of the main criteria for the value of varieties is resistance to harmful viral diseases. The cultivation of such varieties will reduce the cost of producing planting material for small fruit crops of the highest quality categories. In the near future, the most relevant areas for the breeding of small fruit crops will be: breeding for resistance to the most harmful viruses, winter hardiness, increased transportability and long-term post-harvest storage of fruits, suitability for mechanized cultivation, high content of biologically active substances.

## Abstract (ru)

Достижение целевых индикаторов Доктрины продовольственной безопасности (самообеспечение плодами и ягодами не менее 60 %) возможно при объединении компетенций науки и бизнеса. В настоящее время в Государственный реестр селекционных достижений, допущенных к использованию, включены сотни сортов ягодных культур. Отечественными селекционерами получены весомые результаты (79–100 % сортиментa). Лидерами по количеству созданных гибридов и сортов являются ФНЦ Садоводства (Москва) – 101 шт., ФАНЦА (Барнаул) – 85 шт., ФНЦ им. И.В. Мичурина (Мичуринск) – 42 шт. За последние пять лет в государственное сортоиспытание поступило 133 новых сорта традиционных ягодных культур, оригинаторами которых являются научные учреждения, частные компании и лица. Решить проблемы современной селекции и питомниководства, придать импульс развитию отечественного ягодоводства призваны создающиеся на базе ведущих профильных научных учреждений современные селекционно-семеноводческие (питомниководческие) центры (ССЦ). Научно-исследовательские программы ССЦ предполагают комплексный подход, объединяющий знания и возможности ученых разных дисциплин, концентрацию сложной аналитической приборной базы в центры коллективного пользования, использование, наряду с традиционными методами селекции, биотехнологических и молекулярно-генетических исследований. Анализ достижений по ягодоводству в научно-исследовательских учреждениях, подведомственных Министерству науки и высшего образования России, выявил огромный научный потенциал (генетические коллекции, гибридные фонды) для создания конкурентоспособных промышленных сортов и технологий их возделывания путем закладки насаждений сертифицированным в соответствии с международными требованиями посадочным материалом. Информация из литературных источников указывает на один из главных критериев ценности сортов – устойчивость к вредоносным вирусным заболеваниям. Возделывание таких сортов позволит сократить затраты на производство посадочного материала ягодных культур высших категорий качества. В ближайшее время наиболее актуальными направлениями селекции ягодных культур будут: селекция на устойчивость к наиболее вредоносным вирусам, зимостойкость, повышенная транспортабельность и длительное послеуборочное хранение плодов, пригодность к механизированному возделыванию, высокое содержание биологически активных веществ.

## Introduction

Fruit growing production mostly determines the physiological basis of people’s health. Fruits and berries are dietary low-caloric products, rich in easily digested carbohydrates, pectin, organic acids and biologically active substances that include А, В1, В2, В6, Е, K1, С, Р and others, microelements (more than 50 items), anthocyans, flavonoids, tannins and others (Hummer, Barney, 2002; Saveliev et al., 2004; Sedov et al., 2007; Xiao et al., 2017; Mushinsky et al., 2019). Thanks to a wide range of phytonutrients, fruits and berries possess antioxidant, anti-inflammatory, anti-tumor and other treatment-and-prophylactic properties (Landele, 2011).

The consumption of fruits and berries in the Russian Federation is more than 5.0 mln tons or 34 kg per a person at average while the recommended medical norm is 88 kg without citrus fruits and grapes. At the same time, this parameter achieves 50 kg in China, 126 kg in Germany, 135 kg in France, 187 kg in Italy. According to the data of the Ministry of Agriculture of Russia, the record harvest of fruits and berries was gathered in 2019 – 3.5 mln tons, meanwhile domestic production supported only 39.5 % of the medical norm.

To solve the task of providing the population with fruits and berries and the problem of import substitution, one of the most reliable and effective sources of quick increase of the production is the growing of small fruit crops (strawberries, raspberries, black and red currants, gooseberries, honeysuckle and others). Firstly, the areal of their natural growth and industrial production is much wider than the geographical area of fruit cultures. Secondly, small-sized small fruit crops plants characteristically exhibit easy vegetative propagation, quick start of fruiting, early and nonsimultaneous period of fruits ripening (from June to October). Thirdly, high regular crop yield (till 10–15 tons of berries/ha), ecological flexibility, maturity of cultivation technology using mechanical means create cost-effective conditions for their growing (Kazakov et al., 2016).

## Results and discussion

Small fruit crops plantations in Russia occupy nearly 100 thousand ha, 85 % from which belong to private subsidiary plots that are generally the source of self-production. However, they are not fully able to satisfy the demand of population in fresh berries and provide the processing industry with raw materials.

Small fruit growing in Russia can take the leading place by reviving and developing industrial production in close cooperation of science and business thanks to the implementation of new breeding achievements, the use of planting material certified in accordance with the international requirements and modern technologies of cultivation, storage and processing. At present, scientific support of the industry is fulfilled by Federal Horticultural Research Center for Breeding, Agrotechnology and Nursery (FRC of Horticulture, Moscow), Federal Research Center named after I.V. Michurin (FRC named after I.V. Michurin, Michurinsk), Ural Federal Agrarian Research Center of the Ural Branch of the Russian Academy of Sciences (UrFARC UrB RAS, Ekaterinburg), Federal Altai Research Center for Agrobiotechnology (FARCA, Barnaul), All-Russian Research Institute of Fruit Crop Breeding (VNIISPK, Orel Region), North Caucasian Federal Scientific Center of Horticulture, Viticulture, Wine-Making (NCFSCHVW, Krasnodar), Federal Research Center the N.I. Vavilov All-Russian Institute of Plant Genetic Resources (VIR, Saint-Petersburg), Buryat Research Institute of Agriculture (Buryat RIA, Ulan-Ude) and others, as well as specialized departments of institutions of higher education. Meanwhile there is a lack of qualified staff, especially young specialists, both in scientific and production spheres.

The main item of the complex system of small fruit production is variety. The contribution of a variety in increasing the harvest quantity and the quality can be 50–80 %, and the role of genetic-breeding researches will constantly increase (Zhuchenko, 2003; Lugovskoy et al., 2004). Incorrect choice of variety will lead to a decrease in crop yield and cost efficiency, and sometimes to premature death of the plantation and great losses (Knyazev et al., 2012).

At present, dozens of the main small fruit varieties are included in the State Register of Breeding Achievements approved for use in Russia (https://reestr.gossortrf.ru/). Russian crop breeders have received essential results: the proportion of the domestic assortment is 79–100 % depending on the crop. The following institutions created the majority of the varieties: FRC of Horticulture (Moscow) – 102 cvs., FARCA (Barnaul) – 85 cvs., FRC named after I.V. Michurin (Michurinsk) – 42 cvs. (Table 1).

**Table 1.**
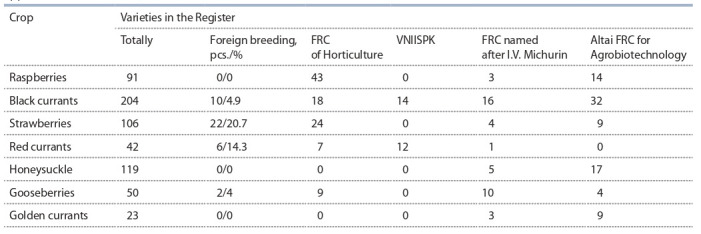
The number of the main small fruit varieties included in the State Register of Breeding Achievements approved for use in Russia, 2020

It should be admitted, that from the enormous number of varieties approved for use, one part was received 30–40 years ago and does not correspond to the consumers’ requirements and modern technologies of cultivation. In this respect, an urgent necessity of revising approaches to the operation of the system of the state crop variety testing has appeared.

Domestic small fruit crops producers have at their disposal varieties of almost all the crops that possess reliable adaptation to environmental conditions, high crop yield and small fruit quality. Based on comprehensive study, the scientists of FRC of Horticulture formed the industrial assortment of small fruit crops recommended for different regions of Russia. However, wide implementation of these varieties is constrained by the absence of a nursery system based on science in Russia. Because of nonobservance of zone cultivation technologies, the biological potential of small fruit crops varieties yield is used only to 20–50 %. Thus, the best varieties of strawberries are able to give 20–25 tons of small fruits from 1 ha in the conditions of high agrotechnique, but in fact the average yield of this crop does not exceed 6 t/ha. The average yield of strawberries varies from 2 to 4 t/ha, while according to the data of the state variety test plots, the potential yield of such varieties as Balzam, Kirzhach, Peresvet and others can achieve 8–10 t/ha.

The use of perspective varieties and the observance of the main technological methods of their cultivation allow to increase the yield essentially. Thus, when cultivating such new primocane varieties of raspberries as Atlant, Zhar-Ptizha, Poklon Kazakovu, Podarok Kashinu in “P(F)H Sychev” of Bryansk Region, the average yield achieved 11–12 t/ha for 3 years with yearly under-winter mowing.

A little portion of small fruit plantations in huge specialized households is mostly connected with high labor intensity of hand harvesting which takes up to 70 % of all expenses (Sazonov, 2006; Kazakov et al., 2009). Depending on the crop, variety or yield this operation requires 200–300 man work units/ha, which is 3–5 times higher than is necessary for fruit crop harvesting. These expenses can be greatly decreased with the help of wide implementation of mechanized harvesting. To achieve it, varieties should correspond to certain requirements (even ripening, easy separation, increased strength and so on). This breeding branch is well developed on black currants. There are nearly 30 varieties of this crop in the State Register of Breeding Achievements of Russia that are suitable for mechanized harvesting (Bagira, Malenky Prints, Mif, Ocharovanie, Orlovskaya Serenada, Orlovsky Vals, Rita, Tamerlan, Charodey, Sharovidnaya, Fortuna and others) (Table 2). The varieties of red currants, raspberries and honeysuckle suitable for machine harvesting have been bred (Bryksin, 2017). Though the list of such varieties is not large, the existing assortment allows to cultivate these crops using totally mechanized technologies. 

**Table 2.**
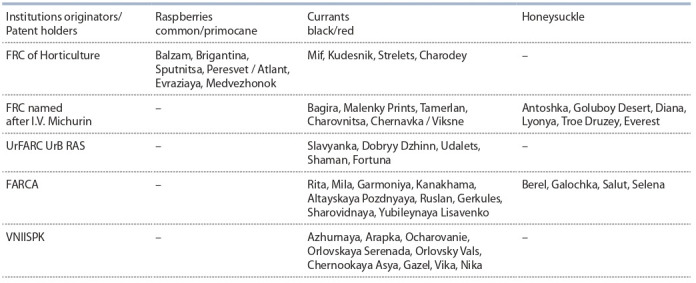
Varieties of the main small fruits crops of Russian breeding recommended for mechanized harvesting

Despite the achieved successes of domestic breeding schools, onrush of modern cultivation technologies, changes of ecological conditions, constant evolution of blasts and diseases require further modernization of assortment. Over the last 5 years, 133 new varieties of the main small fruit crops, the originators of which were not only research centers, but private companies and individuals as well, were applied to the State Variety Commission (Table 3).

**Table 3.**
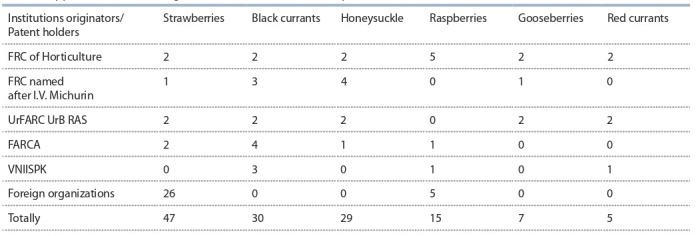
Applications for breeding achievements of small fruit crops submitted in 2016–2020

Along with it the number of applications for the varieties of foreign breeding has increased greatly, especially for the positions where there are gaps and lags of Russian science. Besides, highly marketable varieties of strawberries applicable for industrial cultivation technologies and the use in areas under glass are actively imported. Availability of relatively cheap planting material and the lack of quality seed pieces of domestic varieties facilitate the expanse. 

Analysis of the applications for breeding achievements shows the absence of problems in variety formation of such crops as honeysuckle and black currants. Until recently, honeysuckle was considered as not widespread small fruit crop. The first two varieties (Bakcharskaya and Tomichka) were included in the State Register of Breeding Achievements in 1987. The breeding of this crop develops very quickly. Now, there are 119 varieties and 29 applications for new varieties in the Register. More than that, all of them are domestically bred. Active work in new varieties creation is held mostly in FSUE “Bakcharskoe” (Tomsk region), FRC named after I.V. Michurin, FARCA, Nizhegorodskaya State Agricultural Academy.

The breeding of black currants has been carried out very successfully. 20 breeders in 15 scientific institutions situated in the main cultivation regions of this crop (from Bryansk to Transbaikal) are occupied with this work. Essential bioresource collections and hybrid funds of this crop are created in FRC of Horticulture, VNIISPK, FRC named after I.V. Michurin and UrFARC UrB RAS. These institutions bred productive varieties with high adaptation to cultivation conditions and resistance to American mildew (Orlovskaya Serenada, Orlovsky Vals, Rita, Selechenskaya 2, Strelets, Tamerlan, Sharovidnaya), to currant big bud mite (Dar Smolyaninovoy, Kipiana, Litvinovskaya, Mif, Chudnoe Mgnovenie, Shalunya), and suitable for mechanized cultivation technologies including harvesting (Tamerlan, Rita, Mif, Orlovskaya Serenada, Dobryy Dzhinn and others)

One of the burning issues in blackcurrant cultivation is the absence of varieties immune to the big bud mite. The high degree of mechanization favors the pest dispersal over a plantation and shortens its commercial use by two years on the average (Vityaz et al., 2015).

In the last few years, a tendency of raspberries variety breeding slow-up has been observed. Over the last 5 years, 15 applications for new varieties were submitted to State Variety Commission, from which 5 varieties were of foreign origin. Active breeding work with raspberries is held at Kokinsky base station of FRC of Horticulture. The first domestic raspberries varieties suitable for machine harvesting were bred there; a special branch in breeding – the creation of primocane varieties with prior ripening on one-year shoots – was created there. New large-fruited primocane varieties of raspberries Medvezhonok and Yubileynaya Kulikova are characterized by large biological potential of productivity (2.5–3.0 kg/bush), early ripening, high quality of fruits and do not have foreign analogues with the combination of the same parameters.

Analysis of foreign references points at one of the main criteria of the value of small fruit crops varieties (raspberries, currants, strawberries) – resistance to destructive virus diseases. Cultivation of such varieties allows to decrease the expenses for production of young plants of small fruit crops of high quality. That’s why, in the nearest future, the most relevant areas of the raspberries breeding will be: breeding for resistance to viruses RBDV (raspberries bushy dwarf virus), TBRV (tomato black ring virus), RpRSV (raspberries ring spot virus), ArMV (Arabis mosaic virus), SLRSV (strawberries latent ring spot virus), winter hardiness, increased transportability and long-term post-harvest storage of small fruits, suitability for mechanized cultivation, high content of biologically active substances. 

The assortment of red currants and gooseberries is modernized slowly, which is connected with a limited number of breeders working with these crops. The questions of shoots spinosity and the resistance of many varieties to American mildew (Sphaerotheca) continue to be very important for gooseberries (Kurashov et al., 2019). The cultivation of such varieties will definitely enlarge the interest to the expansion of plantations of such valuable high productive crop.

## Conclusion

Four modern seed production and breeding centers created on the base of leading specialized scientific institutions (FRC of Horticulture, Moscow; FRC named after I.V. Michurin, Michurinsk; NCFSCHVW, Krasnodar; VNIISPK, Orel) have to solve the problems of modern breeding and nursery selection of small fruit crops, to give impetus to the development of domestic small fruit growing. The most important working branch of such centers is the establishment of field repositories that can help objectively evaluate fruits quality and productivity. Combined work of breeders, nurserymen, virologists and other specialists on such plantations will allow to find perspective varieties and clones, to use healthy pollen and to receive parent plants without using expensive and continuous sanitation (Egorov et al., 2020). The research programs of the seed production and breeding centers involve an integrated approach that combines the knowledge and capabilities of researchers from different disciplines, the concentration of a complex analytical instrument base in the Centers of collective use, the use of biotechnological and molecular genetic research along with traditional methods of breeding.

## Conflict of interest

The authors declare no conflict of interest.
